# Survival prediction using the Freiburg index of post-TIPS survival (FIPS) in critically ill patients with acute- on chronic liver failure: A retrospective observational study

**DOI:** 10.3389/fmed.2022.1042674

**Published:** 2022-12-22

**Authors:** Hendrik Luxenburger, Katharina Schmidt, Paul Biever, Alexander Supady, Asieb Sekandarzad, Natascha Roehlen, Marlene Reincke, Christoph Neumann-Haefelin, Michael Schultheiss, Tobias Wengenmayer, Robert Thimme, Dominik Bettinger

**Affiliations:** ^1^Department of Medicine II, Medical Center University of Freiburg, Faculty of Medicine, University of Freiburg, Freiburg im Breisgau, Germany; ^2^IMM-PACT, Medizinische Fakultät, Albert-Ludwigs-Universität Freiburg, Freiburg im Breisgau, Germany; ^3^Interdisciplinary Medical Intensive Care, Faculty of Medicine, University Medical Center–University of Freiburg, Freiburg im Breisgau, Germany; ^4^Berta-Ottenstein-Programme, Faculty of Medicine, University of Freiburg, Freiburg im Breisgau, Germany

**Keywords:** liver cirrhosis, acute-on-chronic liver failure, portal hypertension, intensive and critical care, prognosis

## Abstract

**Background and aim:**

Liver cirrhosis in patients treated in the intensive care unit (ICU) is associated with high mortality. Well established scores are useful to allow for assessment of prognosis and support ICU treatment guidance. However, currently used scoring systems often do not reflect the complexity of critically ill patients. Therefore, we tested the newly developed Freiburg index-of post-TIPS survival (FIPS) score in order to assess its potential role for prognostication of cirrhotic patients in the ICU.

**Methods:**

A total of 310 patients with liver cirrhosis treated in the ICU between 2010 and 2021 were enrolled in this retrospective observational study. Prognostic factors for mortality and 28-day mortality were assessed. Moreover, using c indices the prognostic discrimination of different prognostic scores was analyzed.

**Results:**

The FIPS score allowed to discriminate patients with high ICU mortality and within 28-days after ICU treatment (ICU mortality: 42.2 vs. 59.9%, *p* = 0.008 and 28-day mortality: 43.3 vs. 74.1%, *p* < 0.001). However, the FIPS score in its current composition showed no superior prognostic discrimination compared to other established scores. Multivariable analyses identified the FIPS score (HR 1.25 [1.04–1.49], *p* = 0.015) and lactate at admission (HR 1.07 [1.04–1.09], *p* < 0.001) as significant predictors of ICU mortality. Lactate at admission substantially improved patient risk stratification within each FIPS risk groups.

**Conclusion:**

Similar to other commonly used scores, the FIPS score in its current composition does not allow a sufficiently reliable prognostication of critically ill patients treated in the ICU. However, adding lactate as additional factor to the FIPS score may improve its prognostic ability.

## Introduction

Acute-on-chronic liver failure (ACLF) has been recognized as a distinct syndrome that may develop in approximately 30% of patients with decompensated liver cirrhosis (1). ACLF is characterized by extrahepatic organ failure and associated with a significant increase in short-term mortality (2). Depending on the extent of extrahepatic organ failure, 28-day mortality ranges from 23.3% in patients with ACLF grade I (single organ-failure) up to 75.5% in patients with ACLF grade III (three or more organ failures). Patients with ACLF may require organ support be provided on an intensive care unit (ICU). Mortality in these patients is particularly high, reaching up to 66%, according to recent trials (3, 4). Some studies even report in-hospital mortality as high as 100% for liver cirrhosis requiring ICU treatment (4). These high mortality rates question the utility and value of life-sustaining treatments. Therefore, tools for reliable prognostication are essential for selection of patients and treatment guidance. Previously introduced liver-specific scoring systems, such as the model of end-stage liver disease (MELD), MELD-sodium and Child-Pugh (CP) scores have been developed for prediction of prognosis in non-critically ill patients with liver cirrhosis. However, none of these includes factors of extrahepatic organ function, limiting their ability for prognostication of patients in the ICU. In contrast, the CLIF-C ACLF score has been specifically developed in order to overcome this limitation and incorporates several parameters for the assessment of extrahepatic organ function (5). Moreover, a modification of this score incorporating lactate as an additional factor was proposed (6).

Recently, the Freiburg index of post-TIPS survival (FIPS) has been proposed for prognostication of patients with liver cirrhosis being allocated to implantation of transjugular intrahepatic shunt (TIPS) and has also been validated in different cohorts (7–11). The use of the FIPS outside the TIPS setting, especially in patients with more advanced liver disease and particularly ACLF remains unclear. The aim of this study was to analyze the prognostic value of the FIPS compared to established scores in patients with ACLF treated on the ICU.

## Patients and methods

### Patient selection and follow-up

**FIGURE 1:**
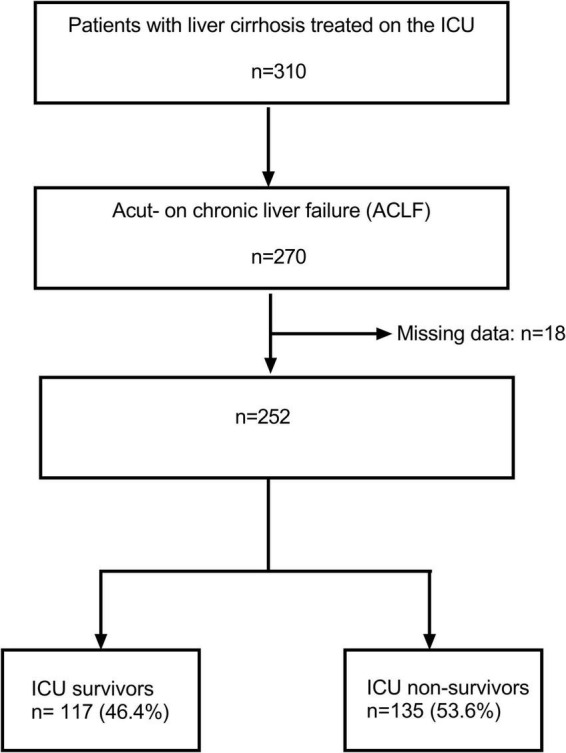
Flow chart for patient selection.

In total, 310 patients with liver cirrhosis who have been treated on the Interdisciplinary Medical Intensive Care Unit (ICU) at the Freiburg University Medical Center from January 1, 2010 through December 31, 2021 were identified by database search (Figure 1). All patients who fulfilled diagnostic criteria for acute-on-chronic liver failure (ACLF) according to the European Foundation for the Study of chronic liver failure (EF CLIF) were selected for analysis (*n* = 270) (2, 12). Subsequently, 18 patients have been excluded due to missing data. Consequently, 252 patients were available for analysis. A total of 93 patients (36.9%) were alive 28 days after ICU treatment and 135 patients (53.6%) died during their stay in the ICU.

### Study endpoints and definitions

Baseline parameters were recorded on admission to the ICU. Laboratory parameters were also assessed at the time of ICU admission. Serum lactate was analyzed with point-of-care testing and values were recorded at admission and after 48 h to calculate the lactate clearance within 48 h as follows:


$$\begin{array}{l} \mathrm{Lactate clearance}\,(\%)\\ =\frac{\begin{array}{l} \mathrm{Lactate}\;\left(\mathrm{at ICU admission}\right)\\ -\mathrm{Lactate}\;(48 hours ater ICU admission) \end{array}}{\mathrm{Lactate}\;\left(\mathrm{at ICU admission}\right)}*100 \end{array}$$

No lactate clearance was defined in values ≤0. All clinical and imaging data were extracted from the electronic patient records. Patients were followed up for 28 days after ICU admission. The primary endpoint was ICU mortality. A 28-day mortality and the course of disease during ICU treatment were assessed as secondary endpoints. The FIPS, MELD, MELD-sodium, CP scores, and CLIF-C ACLF score at the time of ICU admission were calculated for each patient, as reported (7, 13–16). For allocation to the FIPS risk groups, the proposed cut-off of 0.92 was applied (7). Liver cirrhosis was diagnosed by pathognomonic clinical findings in all patients. ACLF was graded according to the european association for the study of the liver (EASL)-CLIF criteria (2, 17). Importantly, the ACLF criteria were introduced in 2013. In all patients that have been included before 2013, data for ACLF diagnosis and staging were available in the electronic medical records.

### Ethics statement

The study was approved by the Freiburg University Ethics Committee (EK 454/19) and is in accordance with the Declaration of Helsinki. Due to the retrospective design of the study informed patient consent was waived. The study was conducted following the STROBE guidelines (18).

### Statistical analyses

The study is a retrospective observational analysis. Continuous variables are expressed as median with interquartile range (25th through 75th percentile). Categorical variables are shown as frequencies and percentages. Group differences were analyzed using Chi-square test or Fisher’s Exact test. For continuous variables, differences were assessed with Wilcoxon rank sum, Kruskal Wallis, Wilcoxon signed rank, and Friedman tests, as appropriate.

Overall survival time (OS) was calculated using the Kaplan-Meier method with death being recorded as event. Differences in survival were assessed using log-rank tests.

Discriminatory performance of the FIPS score in comparison to the CLIF-C ACLF (lactate) score, the MELD (–sodium) and CP score was assessed using Harrell’s C concordance index (c index) using STATA’s somers’ D package. Calibration was assessed by splitting the FIPS score in 10 similar groups and Hosmer–Lemeshow test was applied. Further, calibration was assessed by visual inspection of the Kaplan Meier curves.

In order to analyze prognostic factors, uni- and multivariable Cox regression analyses were performed. Parameters showing *p*-values < 0.1 in the univariable models were entered in the multivariable Cox regression models without further variable selection.

*P*-values < 0.05 were considered significant. Statistical analyses were performed using STATA^®^ (Version 17.0, StataCorp Lp., College Station, TX, USA), SPSS^®^ (Version 29.0, IBM, Armonk, NY, USA) and Prism ^®^ (Version 9.3, GraphPad Software, San Diego, CA, USA).

## Results

### Baseline characteristics of the included patients

Table 1 baseline characteristics of all patients included in this analysis, stratified by ICU survival [survivors (*n* = 117) and non-survivors (*n* = 135), respectively]. The leading etiology of chronic liver disease in this patient cohort was chronic alcohol abuse (50.4%). A total of 42.2% of the ICU non-survivors had alcoholic liver disease compared to 59.8% of the ICU survivors (*p* = 0.006). Gastrointestinal bleeding including variceal bleeding (28.2%), respiratory insufficiency (20.2%) and sepsis (18.3%) were the most common indications for ICU admission. Of note, among ICU non-survivors there were significantly more patients who were admitted to the ICU due to sepsis (24.4 vs. 11.1%, *p* = 0.005). In contrast, in the group of ICU survivors, there were more patients who were admitted to variceal bleeding (19.7 vs. 9.6%, *p* = 0.030). As expected, patients who did not survive ICU treatment needed vasopressor support (98.5 vs. 80.3%, *p* < 0.001), mechanical ventilation (85.9 vs. 55.6%, *p* < 0.001), and continuous renal replacement therapy (CRRT, 43.0 vs. 20.5%; *p* < 0.001) more frequently. In line with these results, 90.1% of the included patients presented with circulatory failure, 69.0% with respiratory failure and 60.7% with renal failure according the CLIF organ failure scoring system. In the ICU non-survivor group, more patients developed renal failure (69.6 vs. 50.4%; *p* = 0.002), respiratory failure (80.7 vs. 55.6%; *p* < 0.001), and circulatory failure (98.5 vs. 80.3%; *p* < 0.001). Overall, 135 patients (53.6%) died during ICU treatment and 28-day mortality was 63.1%. None of the included patients received liver transplantation during the follow-up.

**TABLE 1:** Baseline characteristics of study patients stratified in intensive care unit (ICU) survivors and non-survivors.

	All patients *n* = 252	ICU survivors *n* = 117	ICU non-survivors *n* = 135	*P*-value
Age in years	61 (52–69)	63 (53–72)	60 (52–67)	0.062
Gender (male)	176 (69.8)	88 (75.2)	90 (66.7)	0.272
**Etiology of chronic liver disease**				
Alcohol	127 (50.4)	70 (59.8)	57 (42.2)	0.006
HCV^1^	60 (23.8)	21 (17.9)	39 (28.9)	0.054
HBV^2^	18 (7.1)	7 (6.0)	11 (8.1)	0.626
NAFLD^3^	15 (6.0)	8 (6.8)	7 (5.2)	0.604
Others	32 (12.7)	11 (9.4)	21 (15.6)	0.184
**Reason for ICU admission and organ support**				
Variceal bleeding	36 (14.3)	23 (19.7)	13 (9.6)	0.030
Other GI bleeding	35 (13.9)	19 (16.2)	16 (11.9)	0.365
Sepsis	46 (18.3)	13 (11.1)	33 (24.4)	0.005
Respiratory insufficiency	51 (20.2)	18 (15.4)	33 (24.4)	0.085
Renal failure including electrolyte disturbance	30 (11.9)	19 (16.2)	11 (8.1)	0.053
Loss of consciousness	33 (13.1)	18 (15.4)	15 (11.1)	0.352
Resuscitation	9 (3.6)	3 (2.6)	6 (4.4)	0.510
Others	12 (4.8)	4 (3.4)	8 (5.9)	0.391
Lowest MAP within 48 h (mmHg)	54 (45–60)	57 (49–61)	50 (37–56)	<0.001
Vasopressor support	227 (90.1)	94 (80.3)	133 (98.5)	<0.001
Norepinephrine, peak dose within 48 h (μg/min/kg)	0.40 (0.15–0.62)	0.18 (0.04–0.36)	0.58 (0.40–0.75)	<0.001
Mechanical ventilation	171 (67.9)	65 (55.6)	116 (85.9)	<0.001
CRRT	82 (32.5)	24 (20.5)	58 (43.0)	<0.001
CRRT started on ICU	68 (27.0)	18 (15.4)	50 (37.0)	<0.001
Start of CRRT after admission on ICU (days)	2 (1–5)	1 (0–3)	2 (1–6)	0.023
**Decompensating events**				
No ascites	52 (20.6)	25 (21.4)	27 (20.0)	0.876
Moderate ascites	148 (58.7)	73 (62.4)	75 (55.6)	0.306
Massive ascites	52 (20.6)	19 (16.2)	33 (24.4)	0.120
Spontaneous bacterial peritonitis	50 (19.8)	16 (13.7)	34 (25.2)	0.059
Hepatocellular carcinoma	39 (15.5)	17 (14.5)	22 (16.3)	0.730
**Clinical scores**				
CHILD-Pugh	10 (8–12)	9 (8–11)	11 (9–12)	<0.001
A	9 (3.6)	5 (4.3)	4 (3.0)	0.737
B	88 (34.9)	53 (45.3)	35 (25.9)	0.001
C	155 (61.5)	59 (50.4)	96 (71.1)	0.001
MELD	25 (18–33)	22 (15–27)	26 (21–35)	<0.001
MELD-sodium	27 (20–34)	24 (17–31)	30 (24–36)	<0.001
CLIF-C ACLF	63 (55–73)	58 (52–63)	62 (56–68)	<0.001
FIPS score	1.29 (0.56–2.00)	1.07 (0.38–1.71)	1.49 (0.85–2.16)	0.001
Low risk	90 (35.7)	52 (44.4)	38 (28.1)	0.008
High risk	162 (64.3)	65 (55.6)	97 (71.9)	
**ACLF parameters**				
ACLF Ia	7 (2.8)	7 (6.0)	0	0.004
ACLF Ib	19 (7.5)	17 (14.5)	2 (1.5)	<0.001
ACLF II	90 (35.7)	53 (45.3)	37 (27.4)	0.004
ACLF III	136 (54.0)	40 (34.2)	96 (71.1)	<0.001
CLIF-OF Score	12 (11–14)	11 (10–12)	13 (12–14)	<0.001
Renal failure	153 (60.7)	59 (50.4)	94 (69.6)	0.002
Liver failure	44 (17.5)	14 (12.0)	30 (22.2)	0.045
Coagulation failure	40 (15.9)	15 (12.8)	25 (18.5)	0.231
Brain failure*	35 (13.9)	15 (12.8)	20 (14.8)	0.717
Respiratory failure	174 (69.0)	65 (55.6)	109 (80.7)	<0.001
Circulatory failure	227 (90.1)	94 (80.3)	133 (98.5)	<0.001
**Laboratory parameters**				
WBC (ths/μl)	11.7 (7.1–17.8)	10.7 (6.7–16.5)	12.2 (7.7–18.7)	0.200
Hemoglobin (g/dl)	8.7 (7.4–10.4)	8.7 (7.5–10.5)	8.5 (7.3–10.4)	0.516
Platelets (ths/μl)	95 (59–165)	106 (74–178)	88 (47–135)	0.001
Bilirubin (mg/dl)	3.5 (1.4–8.9)	2.4 (1.3–6.7)	4.3 (1.6–11.1)	0.013
Albumin (g/dl)	2.4 (2.0–2.9)	2.5 (2.1–3.0)	2.3 (1.9–2.9)	0.182
Sodium (mmol/l)	135 (129–139)	136 (131–140)	135 (129–139)	0.379
Creatinine (mg/dl)	1.9 (1.2–3.2)	1.8 (1.1–3.1)	2.1 (1.4–3.2)	0.150
C-reactive protein (ng/ml)	42.0 (18.0–96.0)	33.0 (14.0–76.0)	55.5 (19.3–111.8)	0.009
PCT (pg/ml)	1.5 (0.5–4.5)	0.69 (0.34–2.71)	2.0 (0.8–6.1)	<0.001
Lactate (mmol/l)	3.6 (1.9–8.5)	2.7 (1.6–5.3)	6.4 (2.5–11.0)	<0.001
Lactate clearance within 48 h (%)	21.4 (−18.3 to 54.9)	34 (0.6–62.8)	−0.5 (−50.9 to 36.8)	<0.001

Continuous variables are presented as median with the interquartile range (25. and 75. percentile). Categorial variables are presented as absolute and relative frequencies [*n* (%)].

*Hepatic encephalopathy grade 3–4 according to the West Haven criteria.

### The FIPS score identifies patients with a high mortality on the ICU

**FIGURE 2:**
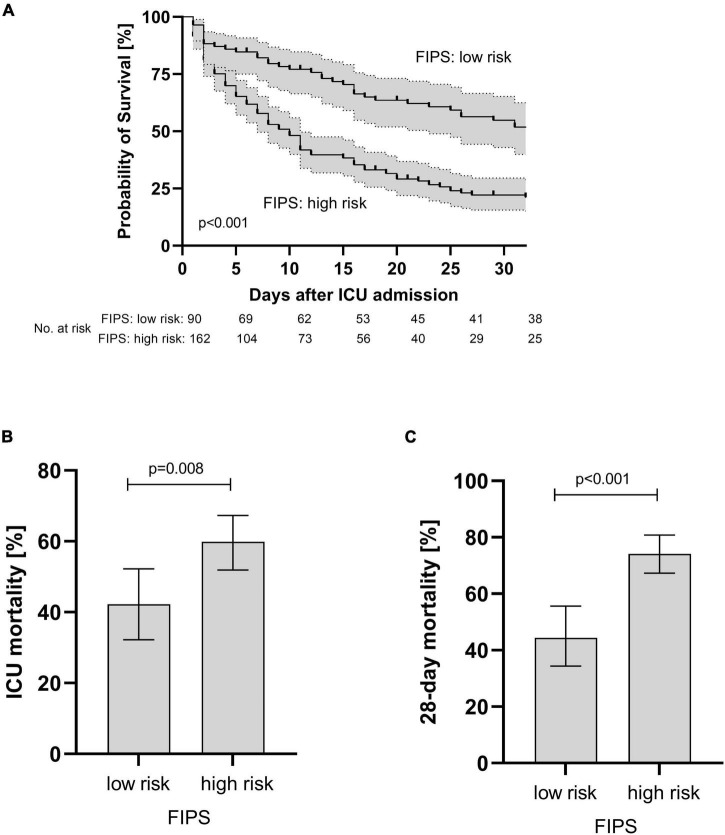
Overall survival **(A)** and ICU **(B)** and 28 day mortality **(C)** of patients with acute-on chronic liver failure (ACLF) stratified according to the FIPS score. Mortality rates are presented as relative frequencies with the corresponding 95% confidence interval. ICU, intensive care unit; FIPS, Freiburg index of post-TIPS survival.

A total of 90 patients (35.7%) were allocated to the FIPS low risk group and 162 patients (64.3%) were classified as FIPS high risk patients. Patients allocated to the FIPS low risk group had a median OS of 31 [10.6–51.4] days compared to 9 [6.9–11.1] days in the high-risk group (*p* < 0.001, Figure 2A). In line with these findings, ICU mortality and 28-day mortality were higher in FIPS high risk patients compared to low risk patients (59.9 vs. 42.2%; *p* = 0.008 and 74.1 vs. 43.3%, *p* < 0.001; Figures 2B, C).

As the FIPS score incorporates serum creatinine levels that may be altered due to renal replacement therapy (RRT), a subgroup analysis was performed excluding patients with RRT initiated before admission to ICU (*n* = 14). Of note, the ability of the FIPS score for prognostic stratification of the patients with regard to OS and ICU and 28-day mortality was confirmed (Supplementary Figures 1, 3 and Supplementary Tables 1, 2).

In order to elaborate the reasons for the higher mortality in the FIPS high risk group in more detail, various factors were assessed and compared between the FIPS low and high-risk group (Table 2). In patients allocated to the FIPS high risk group less often variceal bleeding (9.3 vs. 23.3%, *p* = 0.003), but more frequently infectious complications including sepsis (22.8 vs. 10.0%, *p* = 0.016) and spontaneous bacterial peritonitis (24.1 vs. 12.2%, *p* = 0.008) were the main reason for ICU admission. Patients in the FIPS high risk group had significantly more often advanced ACLF (Table 2). Moreover, a significantly higher proportion of patients in the FIPS high risk patients needed CRRT on ICU compared to the FIPS low risk patients (33.3 vs. 15.6%, *p* = 0.003).

**TABLE 2:** Comparison of patients in the Freiburg index of post-TIPS survival (FIPS) low and high risk group.

	FIPS low risk group *n* = 90	FIPS high risk group *n* = 162	*P*-value
**Etiology of chronic liver disease**			
Alcohol	58 (64.4)	69 (42.6)	0.001
HCV^1^	21 (23.3)	39 (24.1)	0.999
HBV^2^	8 (8.9)	10 (6.2)	0.450
NAFLD^3^	3 (3.3)	12 (7.4)	0.269
Others	0	32 (19.8)	<0.001
**Reason for ICU admission and organ support**			
Variceal bleeding	21 (23.3)	15 (9.3)	0.003
Other GI bleeding	14 (15.6)	21 (13.0)	0.704
Sepsis	9 (10.0)	37 (22.8)	0.016
Respiratory insufficiency	20 (22.2)	31 (19.1)	0.624
Renal failure including electrolyte disturbance	6 (6.7)	24 (14.8)	0.068
Loss of consciousness	10 (11.1)	23 (14.2)	0.562
Resuscitation	6 (6.7)	3 (1.9)	0.073
Others	4 (4.4)	8 (4.9)	0.999
Lowest MAP within 48 h (mmHg)	56 (46–61)	53 (45–57)	0.034
Vasopressor support	82 (91.1)	145 (89.5)	0.827
Norepinephrine, peak dose within 48 h (μg/min/kg)	0.27 (0.08–0.59)	0.45 (0.18–0.63)	0.115
Mechanical ventilation	65 (72.2)	106 (65.4)	0.325
CRRT	19 (21.1)	63 (38.9)	0.005
CRRT started on ICU	14 (15.6)	54 (33.3)	0.003
**Decompensating events**			
No ascites	21 (23.3)	31 (19.1)	0.516
Moderate ascites	57 (63.3)	91 (56.2)	0.288
Massive ascites	12 (13.3)	40 (24.7)	0.035
Spontaneous bacterial peritonitis	11 (12.2)	39 (24.1)	0.008
Hepatocellular carcinoma	12 (13.3)	27 (16.7)	0.587
**ACLF parameters**			
ACLF Ia	4 (4.4)	3 (1.9)	0.427
ACLF Ib	11 (12.2)	8 (4.9)	0.046
ACLF II	50 (55.6)	40 (24.7)	<0.001
ACLF III	25 (27.8)	111 (68.5)	<0.001
CLIF-OF Score	11 (11–12)	13 (11–14)	<0.001
Renal failure	30 (33.3)	123 (75.9)	<0.001
Liver failure	2 (2.2)	42 (25.9)	<0.001
Coagulation failure	6 (6.7)	34 (21.0)	0.004
Brain failure*	11 (12.2)	24 (14.8)	0.704
Respiratory failure	63 (70.0)	111 (68.5)	0.887
Circulatory failure	82 (91.1)	145 (89.5)	0.827

Continuous variables are presented as median with the interquartile range (25. and 75. percentile). Categorial variables are presented as absolute and relative frequencies [*n* (%)].

*Hepatic encephalopathy grade 3–4 according to the West Haven criteria.

### Prognostic discrimination of the FIPS score compared to the Child- Pugh-, MELD (sodium) and CLIF-C ACLF score

In order to assess prognostic discrimination capacity of the FIPS score compared to other established scores for patients with ACLF treated on ICU, c indices for ICU mortality and 28-day mortality were calculated (Table 3). The c index of the FIPS score for ICU mortality and 28-day mortality was 0.619 and 0.640 and, thus, not superior to the CLIF-C ACLF (0.584 [*p* = 0.238] and 0.626 [*p* = 0.573]), the MELD (0.590 [*p* = 0.166] and 0.629 [*p* = 0.520]), the MELD-sodium (0.585 [*p* = 0.128] and 0.626 [*p* = 0.346]), and the CP score (0.652 [*p* = 0.281] and 0.657 [*p* = 0.491]). However, the modified CLIF-C ACLF lactate score showed prognostic discrimination superior to the FIPS score (0.688 [*p* = 0.018] and 0.708 [*p* = 0.004]) indicating the prognostic relevance of lactate in these critically ill patients. In summary, the FIPS score does not show a superior prognostic discrimination capacity compared to the previously established liver-related prognostic scores in critical ill patients with ACLF.

**TABLE 3:** Summary of the c index of the FIPS score compared to the CLIF C ACLF (lactate), model of end-stage liver disease (MELD) (–sodium), and CP^4^ score.

	FIPS^1^ c index [95% CI^4^]	CLIF C ACLF^2^ c index [95% CI]	CLIF C ACLF lactate c index [95% CI]	MELD^3^ c index [95% CI]	MELD sodium c index [95% CI]	CP^5^ c index [95% CI]
ICU mortality	0.619 [0.559–0.679]	0.584 [0.518–0.649]	0.688 [0.612–0.741]	0.590 [0.528–0.653]	0.585 [0.523–0.647]	0.652 [0.595–0.709]
*p*-values vs. FIPS	–	0.238	0.018	0.166	0.128	0.281
28-day mortality	0.640 [0.591–0.689]	0.626 [0.577–0.675]	0.708 [0.665–0.751]	0.629 [0.580–0.678]	0.623 [0.573–0.673]	0.657 [0.611–0.703]
*p*-values vs. FIPS	–	0.573	0.004	0.520	0.346	0.491

^1^FIPS, Freiburg index of post-TIPS survival; ^2^ACLF, acute-on chronic liver failure; ^3^MELD, model of end-stage liver disease; ^4^95% CI, 95% confidence interval; ^5^CP, Child-Pugh score.

### Calibration of the FIPS score

The Hosmer-Lemeshow test confirmed similar observed and predicted ICU- and 28-day mortality across the stratified groups of the FIPS score (χ^2^ = 7.65, *p* = 0.4680 for ICU mortality; χ^2^ = 7.66, *p* = 0.4675 for 28-day mortality). In line with these results, the Kaplan Meier curves comparing observed vs. predicted survival showed acceptable calibration of the FIPS score (Supplementary Figure 2).

### Prognostic factors for ICU and 28-day mortality

**FIGURE 3:**
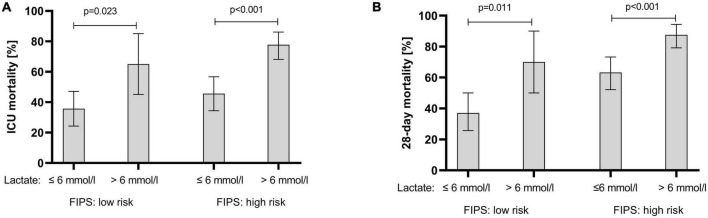
ICU mortality **(A)** and 28-day mortality **(B)** of acute-on chronic liver failure (ACLF) patients stratified according the FIPS risk groups in combination with lactate at ICU admission. Mortality rates are presented as relative frequencies with the corresponding 95% confidence interval. ICU, intensive care unit; FIPS, Freiburg index of post-TIPS survival.

In an attempt to determine further prognostic factors for ICU mortality a multivariable Cox regression model revealed that the FIPS score (HR 1.25 [1.04–1.49], *p* = 0.015) and lactate (HR 1.07 [1.04–1.09], *p* < 0.001) were significant and independent prognostic factors for ICU mortality in patients with ACLF treated on the ICU (Table 4). Extending the observation period to 28 days after ICU admission, the FIPS score (HR 1.44 [1.19–1.73], *p* < 0.001) and lactate (HR 1.07 [1.05–1.09], *p* < 0.001) remained statistically significant and independent prognostic factors (Table 5). The combination of the FIPS score and lactate at admission substantially improved patient risk stratification. Using a lactate cut-off of 6 mmol/l (as determined by receiver operating characteristic (ROC) analysis and the Youden index), the addition of lactate allows the identification of patients with an impaired prognosis within the FIPS risk groups (Figure 3).

**TABLE 4:** Uni- and multivariable Cox regression model for prognostic factors for intensive care unit (ICU) mortality in patients with acute-on chronic liver failure (ACLF).

Parameter	Univariable model				Multivariable model			
	β^1^	HR^2^	95% CI^3^	*P*-value	β	HR	95% CI	*P*-value
Variceal bleeding	–0.25	0.78	0.44–1.38	0.387				
Sepsis	0.20	1.22	0.83–1.82	0.314				
Vasopressor support	1.31	3.71	0.91–15.10	0.067	0.94	2.56	0.63–10.45	0.192
Mechanical ventilation	0.19	1.22	0.75–1.98	0.429				
CRRT start on ICU	–0.11	0.89	0.63–1.28	0.534				
Platelets	–0.002	0.99	0.98–1.00	0.027	–0.001	0.99	0.98–1.01	0.193
C-reactive protein	–0.001	0.99	0.98–1.01	0.237				
Procalcitonin	–0.002	0.99	0.99–1.04	0.485				
Lactate	0.07	1.07	1.05–1.10	<0.001	0.07	1.07	1.04–1.09	<0.001
FIPS^4^ score	0.29	1.33	1.13–1.57	<0.001	0.22	1.25	1.04–1.49	0.015

^1^β, regression coefficient; ^2^HR, hazard ratio; ^3^95% CI, 95% confidence interval; ^4^FIPS, Freiburg index of post-TIPS survival.

**TABLE 5:** Uni- and multivariable Cox regression model for prognostic factors 28-day mortality in patients with acute-on chronic liver failure (ACLF) treated on the intensive care unit (ICU).

Parameter	Univariable model				Multivariable model			
	β^1^	HR^2^	95% CI^3^	*P*-value	β	HR	95% CI	*P*-value
Variceal bleeding	−0.50	0.61	0.37–1.00	0.052	−0.434	0.65	0.38–1.10	0.109
Sepsis	0.38	1.46	1.01–2.11	0.042	0.03	1.03	0.70–1.52	0.883
Vasopressor support	0.99	2.68	1.32–5.45	0.007	0.58	1.78	0.81–3.92	0.153
Mechanical ventilation	0.57	1.76	0.123–2.53	0.002	0.42	1.51	0.99–2.33	0.059
CRRT start on ICU	0.43	1.54	1.11–2.13	0.009	0.03	1.03	0.70–1.05	0.898
Platelets	−0.54	0.58	0.41–0.83	0.002	−0.001	0.99	0.98–1.00	0.335
C-reactive protein	0.001	1.00	0.99–1.01	0.393				
Procalcitonin	−0.001	0.999	0.98–1.01	0.821				
Lactate	0.09	1.09	1.07–1.11	<0.001	0.07	1.07	1.05–1.09	<0.001
FIPS^4^ score	0.40	1.49	1.27–1.74	<0.001	0.36	1.44	1.19–1.73	<0.001

^1^β, regression coefficient; ^2^HR, hazard ratio; ^3^95% CI, 95% confidence interval; ^4^FIPS, Freiburg index of post-TIPS survival.

## Discussion

Liver cirrhosis is associated with high mortality especially in patients admitted to the ICU (3, 4). Reliable prognostication including prediction of mortality risk is very important for the management of these vulnerable patients. Our data demonstrate that the FIPS score may help to identify patients at high risk of dying during ICU treatment. ICU mortality in patients with a high FIPS score was significantly higher than in patients with a low FIPS score (59.9 vs. 42.2%, *p* = 0.008). Extending the observation period to 28 days after admission to ICU, the differences in mortality between FIPS low and high scoring patients was even larger (74.1 vs. 43.3% *p* < 0.001) indicating that the FIPS score may also have a prognostic value even for extended observation periods after ICU discharge.

However, in direct comparison to previously established scores [MELD, MELD sodium, Child-Pugh, CLIF C ACLF (lactate) scores], the FIPS score is not superior in prognostic discrimination of critically ill patients with liver cirrhosis. Several possible reasons may explain the limited prognostic capacity of the FIPS score in these patients. First, the parameters included in the FIPS score are similar to those considered in the other scoring systems and probably they may not sufficiently represent the full complexity of ACLF. Recently, generalized inflammatory response has been recognized as an important factor in the emergence and deterioration of ACLF (19), addition of inflammatory parameters to the FIPS score may increase its prognostic value. However, our multivariable regression analyses do not support the inclusion of inflammatory parameters into FIPS score. Therefore, this aspect should be further evaluated in larger cohorts.

Another important drawback of the FIPS score limiting its use for critically ill patients may be the inclusion of serum creatinine. In patients receiving CRRT creatinine does not reliably describe renal function. Therefore, we performed a subgroup analysis excluding patients who were already on RRT before admission to the ICU. After exclusion of these patients, the prognostic discriminatory capacity of the FIPS score was confirmed (Supplementary Figure 1).

Finally, albumin plays an important role in the management and assessment of critically ill patients in the ICU. Data on hypoalbuminemia in cirrhotic patients suggest increased mortality in non-critically ill patients on the liver transplant waiting list (20) and a general association with a poor clinical outcome in critically ill patients (21). Therefore, inclusion of albumin in prognostic scores for critically ill patients may be important, as shown by a modification of the MELD score resulting in improved mortality prediction for patients waiting for liver transplantation (22).

But, most critically ill patients show reduced albumin levels and therefore its prognostic capacity may be reduced. Moreover, critically ill cirrhotic patients often receive albumin substitution, e.g., after large-volume paracenteses or for the management of systemic inflammation (23, 24). Therefore, serum albumin values may show significant fluctuation and may be highly influenced by the iatrogenic administration of albumin. This represents a major bias for interpretation of the prognostic relevance of albumin in these patients and may be another important reason for the FIPS score not being superior in prognostication compared to the other established scoring systems.

Our multivariable regression models suggest that that the addition of blood lactate levels might increase the prognostic accuracy of the FIPS score. Generally, lactate and lactate clearance are well established predictors of outcome in critically ill patients on the ICU (25–27). Furthermore, lactate is known to be a relevant prognostic marker for short-term mortality in patients with liver cirrhosis (6). Recent studies have attempted to improve the MELD score and the CLIF-C-ACLF score by adding lactate and have thus achieved better prognostic accuracy compared to the original scores (6, 28). Of note, since lactate levels may fluctuate over the time, the determination of lactate at a single time point may not be sufficient (6). Interestingly, there was no significant superiority of lactate determination in addition to the FIPS at time of admission versus lactate clearance in different regression models (Supplementary Data). Therefore, lactate at admission was used for further risk stratification. Within the FIPS risk groups, the addition of lactate levels helped identification of patients with the highest risk of mortality in the ICU in each group. In conclusion, the addition of lactate to the FIPS score may increase its prognostic capacity. Regrettably, our sample size was too small to assess a modified FIPS-lactate-score for patients with ACLF treated in the ICU. Therefore, further studies with larger sample sizes and internal and external validation cohorts are needed.

Moreover, dynamic assessment of the FIPS score during the course of ICU treatment may be of prognostic impact and may increase its prognostic accuracy. As albumin was only assessed at baseline, we were not able to analyze the FIPS dynamics during ICU treatment. Therefore, this relevant topic should be addressed in further studies.

An important limitation of our study with respect to generalization of our results, is that 69.0% of our patients presented with respiratory failure and even 90.1% with circulatory failure. Previous studies including ACLF patients only reported few patients with respiratory failure (10–16%) (29–31). In line with this observation, the median CLIF C ACLF score in our cohort was significantly higher than in previously reported cohorts (63 vs. 42.6%) indicating a very selected subgroup of patients with ACLF (31). Further, another major limitation of cour study is due to the retrospective design. Patient inclusion was not consecutive and therefore an inherent selection bias cannot be completely ruled out.

Available established scores do not reflect the complexity of critically ill patients with liver cirrhosis in the ICU. However, the FIPS score in its current composition is not superior to these scoring systems and currently not recommended as an alternative. Adding lactate as an additional parameter to a revised FIPS score may improve its accuracy in critically ill patients with ACLF in the ICU. This hypothesis should be assessed in future studies.

## Data availability statement

The raw data supporting the conclusions of this article will be made available by the authors, without undue reservation.

## Ethics statement

The studies involving human participants were reviewed and approved by Ethics Committee of the University of Freiburg, number: EK 454/19. The patients/participants provided their written informed consent to participate in this study.

## Author contributions

DB, HL, and PB: design of the study. DB, HL, KS, NR, and MR: acquisition of data. DB, HL, and KS: analysis and interpretation of the data. DB: statistical analyses and consulting. DB and HL: drafting the manuscript. TW, AS, AsS, MS, CN-H, and RT: revision for important intellectual content. All authors approved the final version of the article.
