# Supplementary material for: Survival prediction using the Freiburg index of post-TIPS survival (FIPS) in critically ill patients with acute- on chronic liver failure: A retrospective observational study
Source: Front Med (Lausanne). 2022 Dec 22;9:1042674. doi: 10.3389/fmed.2022.1042674 (PMC9812953; doi:10.3389/fmed.2022.1042674)
Supplement: Supplementary file 1 [file Data_Sheet_1.pdf]

Supplementary data

Survival prediction using the *Freiburg index of post-TIPS survival* (FIPS) in critically ill patients with acute- on chronic liver failure

Hendrik Luxenburger, Katharina Schmidt, Paul Biever, Alexander Supady, Asieb Sekandarzad, Natascha Roehlen, Marlene Reincke, Christoph Neumann-Haefelin, Michael Schultheiß, Robert Thimme, Tobias Wengenmayer and Dominik Bettinger

Suppl. Figure 1: Overall survival (A) and ICU (B) and 28 day mortality (C) of patients with ACLF without initiation of CRRT during ICU treatment…………………………………………………………………….……………………..II

Suppl. Figure 2: Calibration of the FIPS……………………………………………….III

Comparison of the c index of the Cox regression models including the FIPS score and lactate at ICU admission vs. lactate clearance within 48 hours……………….………IV

Influence of CRRT on lactate and lactate clearance……………………………………IV

Suppl. Figure 1

Suppl. Figure 1: Overall survival (A) and ICU (B) and 28 day mortality (C) of patients with ACLF without initiation of CRRT during ICU treatment. Mortality rates are presented as relative frequencies with the corresponding 95% confidence interval.

Abbreviations: ICU, intensive care unit; FIPS, Freiburg index of post-TIPS survival, CRRT, continuous renal replacement therapy

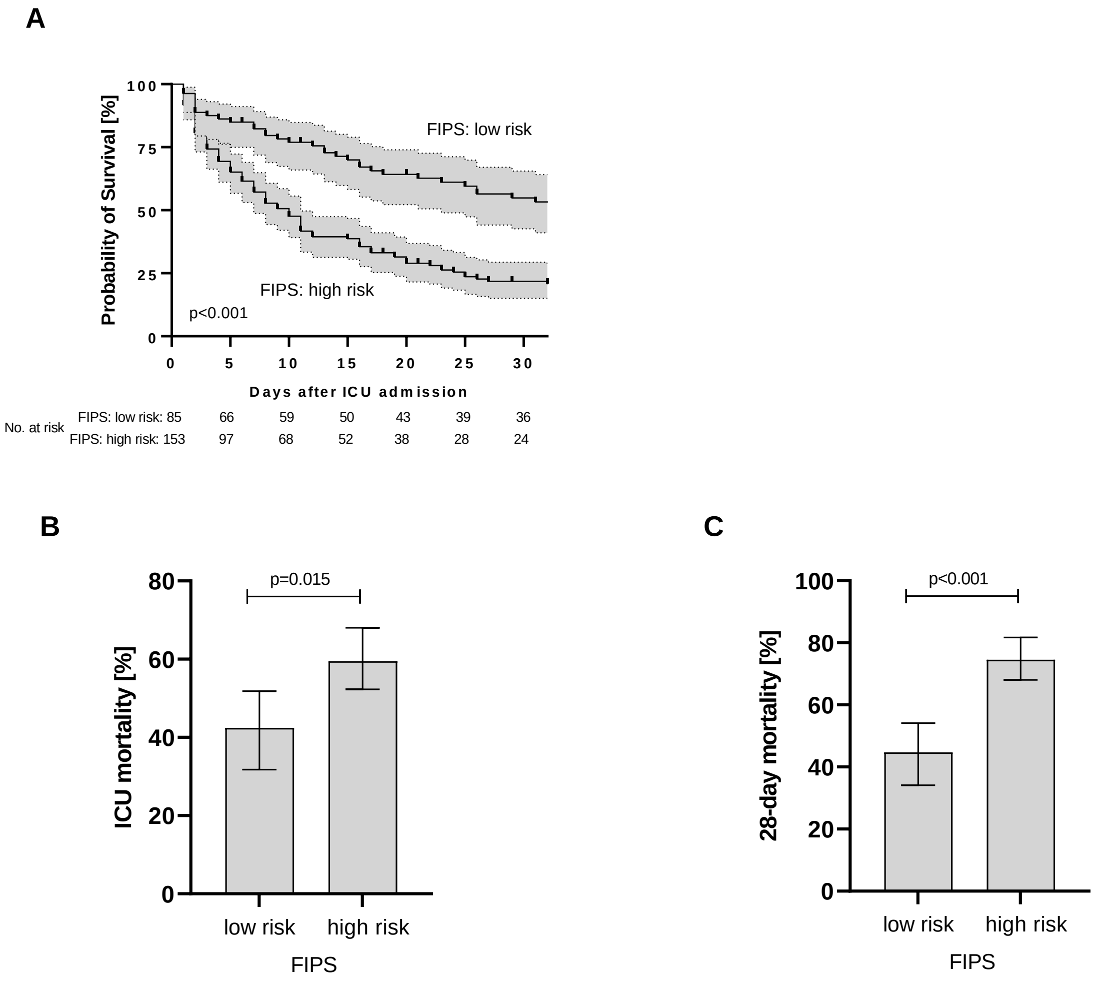

<u>Suppl. Figure 2</u>

Suppl. Figure 1: Calibration of the FIPS.
Calibration plot showing the observed (solid line) vs. predicted (dashed line) 28-day survival in the validation set.

Abbreviations: FIPS, Freiburg index of post-TIPS survival

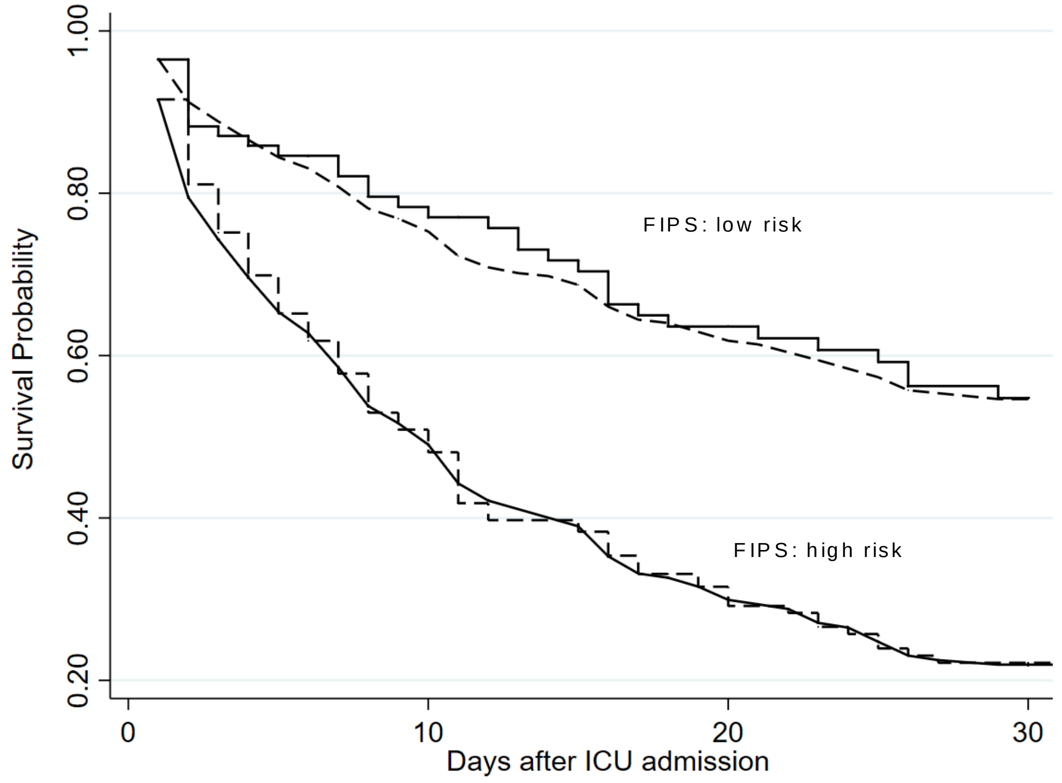

Comparison of the c index of the Cox regression models including the FIPS score and lactate at ICU admission vs. lactate clearance within 48 hours.

a) **ICU mortality**

The Cox regression model including the FIPS score (low vs.high risk group) and lactate at ICU admission showed a c index of 0.690 [0.630-0.750] compared to 0.722 [0.660-0.784] of the model including the FIPS risk groups and lactate clearance within 48 hours (p=0.380).

b) **28- day mortality**

The Cox regression model including the FIPS score (low vs.high risk group) and lactate at ICU admission showed a c index of 0.700 [0.651-0.748] compared to 0.693 [0.642-0.7545 of the model including the FIPS risk groups and lactate clearance within 48 hours (p=0.831).

Influence of CRRT on lactate and lactate clearance

CRRT may have a significant impact on lactate and lactate clearance and may therefore affect the prognostic value of lacate and lactate clearance. Linear regression analysis did not show a significant impact on lactate clearance in our cohort (regression coefficient: 7.978; p=0.727). Further, lactate at admission (3.53 [1.94-7.76] vs. 3.82 [1.96-8.73] mmol/l; p=0.844) and lactate after 48 hours (2.16 [1.58-6.59] vs. 2.34 [1.51-6.72] mmol/l; p=0.857) was similar in patients with and without CRRT. Further, lactate clearance was not significantly different in patients with and without CRRT (**Suppl. figure 3**) .

Suppl. Figure 3

Suppl. Figure 3: Impact of CRRT on lactate and lactate clearance

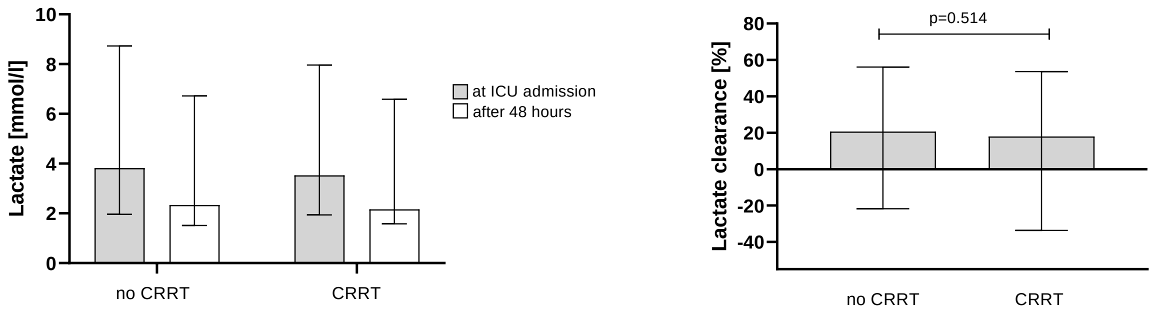

As CRRT may influence lactate clearance, patients were stratified according to CRRT treatment. Cox models including the FIPS score and lactate or lactate clearance were calculated and the c indices were assessed (**Suppl. table 1,2**).

Suppl. table 1

**c index ICU mortality**	**FIPS+lactate**	**FIPS+lactate clearance**	**P value**
**CRRT**	0.716 [0.634-0.787]	0.682 [0.592-0.772]	0.682
**No CRRT**	0.724 [0.653-0.795]	0.746 [0.668-0.825]	0.561

Suppl. table 2

**c index 28-day mortality**	**FIPS+lactate**	**FIPS+lactate clearance**	**P value**
**CRRT**	0.711 [0.642-0.780]	0.692 [0.541-0.717]	0.182
**No CRRT**	0.712 [0.653-0.770]	0.684 [0.619-0.749]	0.385
