# Supplementary material for: Survival prediction using the Freiburg index of post-TIPS survival (FIPS) in critically ill patients with acute- on chronic liver failure: A retrospective observational study
Source: Front Med (Lausanne). 2022 Dec 22;9:1042674. doi: 10.3389/fmed.2022.1042674 (PMC9812953; doi:10.3389/fmed.2022.1042674)
Supplement: Supplementary file 2 [file Table_1.docx]

**Supplementary data**

**Survival prediction using the *Freiburg index of post-TIPS survival* (FIPS) in critically ill patients with acute- on chronic liver failure**

Hendrik Luxenburger, Katharina Schmidt, Paul Biever, Alexander Supady, Asieb Sekandarzad, Natascha Roehlen, Marlene Reincke, Christoph Neumann-Haefelin, Michael Schultheiß, Robert Thimme, Tobias Wengenmayer and Dominik Bettinger

Suppl. Figure 1: Overall survival (A) and ICU (B) and 28 day mortality (C) of patients with ACLF without initiation of CRRT during ICU treatment……………………………………………………………….……………………..II

Suppl. Figure 2: Calibration of the FIPS………………………………………………….III

Comparison of the c index of the Cox regression models including the FIPS score and lactate at ICU admission vs. lactate clearance within 48 hours………………..………IV

Influence of CRRT on lactate and lactate clearance……………………………………IV

**Suppl. Figure 1**

**Suppl. Figure 1: Overall survival (A) and ICU (B) and 28 day mortality (C) of patients with ACLF without initiation of CRRT during ICU treatment.** Mortality rates are presented as relative frequencies with the corresponding 95% confidence interval.

*Abbreviations: ICU, intensive care unit; FIPS, Freiburg index of post-TIPS survival, CRRT, continuous renal replacement therapy*

**Suppl. Figure 2**

**Suppl. Figure 2: Calibration of the FIPS.**

Calibration plot showing the observed (solid line) vs. predicted (dashed line) 28-day survival in the validation set.

*Abbreviations: FIPS, Freiburg index of post-TIPS survival*

**Comparison of the c index of the Cox regression models including the FIPS score and lactate at ICU admission vs. lactate clearance within 48 hours.**

1. **ICU mortality**

The Cox regression model including the FIPS score (low vs.high risk group) and lactate at ICU admission showed a c index of 0.690 [0.630-0.750] compared to 0.722 [0.660-0.784] of the model including the FIPS risk groups and lactate clearance within 48 hours (p=0.380).

1. **28- day mortality**

The Cox regression model including the FIPS score (low vs.high risk group) and lactate at ICU admission showed a c index of 0.700 [0.651-0.748] compared to 0.693 [0.642-0.7545 of the model including the FIPS risk groups and lactate clearance within 48 hours (p=0.831).

**Influence of CRRT on lactate and lactate clearance**

CRRT may have a significant impact on lactate and lactate clearance and may therefore affect the prognostic value of lacate and lactate clearance. Linear regression analysis did not show a significant impact on lactate clearance in our cohort (regression coefficient: 7.978; p=0.727). Further, lactate at admission (3.53 [1.94-7.76] vs. 3.82 [1.96-8.73] mmol/l; p=0.844) and lactate after 48 hours (2.16 [1.58-6.59] vs. 2.34 [1.51-6.72] mmol/l; p=0.857) was similar in patients with and without CRRT. Further, lactate

clearance was not significantly different in patients with and without CRRT (**Suppl. figure 3**) .

**Suppl. Figure 3**

**Suppl. Figure 3: Impact of CRRT on lactate and lactate clearance**

As CRRT may influence lactate clearance, patients were stratified according to CRRT treatment. Cox models including the FIPS score and lactate or lactate clearance were calculated and the c indices were assessed (**Suppl. table 1,2**).

**Suppl. table 1**

| c index  ICU mortality | FIPS+lactate | FIPS+lactate clearance | P value |
| --- | --- | --- | --- |
| CRRT | 0.716 [0.634-0.787] | 0.682 [0.592-0.772] | 0.682 |
| No CRRT | 0.724 [0.653-0.795] | 0.746 [0.668-0.825] | 0.561 |

**Suppl. table 2**

| c index  28-day mortality | FIPS+lactate | FIPS+lactate clearance | P value |
| --- | --- | --- | --- |
| CRRT | 0.711 [0.642-0.780] | 0.692 [0.541-0.717] | 0.182 |
| No CRRT | 0.712 [0.653-0.770] | 0.684 [0.619-0.749] | 0.385 |
